# Are Circulating Cytokines Reliable Biomarkers for Amyotrophic Lateral Sclerosis?

**DOI:** 10.3390/ijms20112759

**Published:** 2019-06-05

**Authors:** Laura Moreno-Martinez, Ana Cristina Calvo, María Jesús Muñoz, Rosario Osta

**Affiliations:** Laboratory of Genetics and Biochemistry (LAGENBIO), Faculty of Veterinary-IIS Aragón, IA2-CITA, CIBERNED, University of Zaragoza, Miguel Servet 177, 50013 Zaragoza, Spain; lauramm@unizar.es (L.M.-M.); mjmunoz@unizar.es (M.J.M.); osta@unizar.es (R.O.)

**Keywords:** cytokines, biomarkers, amyotrophic lateral sclerosis, blood, plasma, serum, cerebrospinal fluid, diagnosis, prognosis, inflammation

## Abstract

Amyotrophic lateral sclerosis (ALS) is a fatal neurodegenerative disease that has no effective treatment. The lack of any specific biomarker that can help in the diagnosis or prognosis of ALS has made the identification of biomarkers an urgent challenge. Multiple panels have shown alterations in levels of numerous cytokines in ALS, supporting the contribution of neuroinflammation to the progressive motor neuron loss. However, none of them is fully sensitive and specific enough to become a universal biomarker for ALS. This review gathers the numerous circulating cytokines that have been found dysregulated in both ALS animal models and patients. Particularly, it highlights the opposing results found in the literature to date, and points out another potential application of inflammatory cytokines as therapeutic targets.

## 1. Introduction

Amyotrophic lateral sclerosis (ALS) is one of the most serious motor neuron diseases, and the most common adult motor neuron disease. It is characterized by loss of the cortex, brainstem, and spinal cord motor neurons (MNs), leading to muscle paralysis, and finally premature death due to respiratory failure within 2–5 years after diagnosis. Unfortunately, to date, no effective therapies able to cure the disease are available. More than 90% of ALS cases are sporadic with unknown causes. On the other hand, around 10% of ALS patients have a family history, involving mutations in a number of genes, such as Cu^2+^/Zn^2+^ superoxide dismutase 1 (SOD1), TAR DNA binding protein 43 (TDP-43), fused in sarcoma (FUS) and chromosome 9 open reading frame 72 (C9ORF72) repeat expansions [1]. These mutations found in ALS have allowed the development of animal models that are helpful in the study of this disease. One of the best characterized animal models for ALS is the SOD1G93A mouse model, which carries a G93A mutation (substitution of Glycine to Alanine at codon 93) in the human SOD1 gene, and presents both clinical and pathological characteristics of ALS patients [2].

The diagnosis of ALS is based on clinical tests and electrophysiological studies [3]. However, there is not a definitive diagnostic test for ALS, and the difficulty to reach it leads to the delay of diagnosis up to one year after the onset of symptoms. In addition, although the ALS Functional Rating Scale–Revised (ALSFRS-R) can functionally measure ALS progression [4], the prognosis in each patient still remains uncertain and challenging to anticipate. 

Numerous mechanisms have been proposed to explain the degeneration of motor neurons, including misfolded protein aggregation and impaired degradation, glutamate excitotoxicity, increased oxidative stress, neuroinflammation and mitochondrial dysfunction [5]. However, the cause of motor neuron degeneration in ALS is still unclear.

## 2. The Necessity of Identifying Biomarkers for ALS

There is an imperious need to identify biomarkers that can help diagnose the disease at earlier stages and also to predict the course of the disease that allow following of more accurate therapeutic strategies. The ideal biomarker should be sensitive enough to diagnose ALS at the pre-symptomatic stage; specific for ALS and able to discriminate from other clinically similar neurodegenerative diseases; able to predict the progression of the disease in each patient; and easily accessible and applicable for all patients, despite their physical condition [6]. Although the origin of ALS remains unknown, multiple panels of biomarkers have been described in ALS patients and murine models to explain the progressive motor neuron loss and muscle atrophy, including imaging, electrophysiological and wet biomarkers [7]. 

Neuroimaging biomarkers allow a faithful visualization of the structural alterations happening in the tissue of study when comparing them with healthy tissues. Different brain imaging techniques have been used to detect these pathological changes in ALS patients, such as magnetic resonance imaging (MRI), magnetic resonance spectroscopy (MRS), diffusion tensor imaging (DTI) and positron emission tomography (PET) [8,9]. As a result of using these techniques, some pathological alterations regarding cortical atrophy, neuronal integrity and brain white matter abnormalities have been positively correlated with progression of the disease or resulted helpful in the diagnosis [10,11,12,13]. Although some promising imaging biomarkers have been found, none of them is fully sensitive and specific enough for ALS diagnosis or prognosis. 

Numerous neurophysiologic biomarkers have been identified and proposed as biomarkers that help in the early diagnosis and monitoring of the progression of the disease [14]. Transcranial magnetic stimulation (TMS) technique can detect cortical hyperexcitability at early stages of the disease, which has been correlated with upper motor neuron dysfunction [15]. On the other hand, the course of ALS can be monitored by assessing lower motor neuron dysfunction using electrical impedance myography, axonal excitability testing, the motor unit number index and muscle ultrasonography [15,16,17]. However, despite the potential neurophysiological biomarkers found so far in assessing progression and early diagnosis, further studies should be conducted involving larger ALS cohorts [15]. 

Multiple molecular markers have been described in cerebrospinal fluid (CSF), plasma, serum, and even urine and saliva. Unlike CSF, the other fluids possess the advantage of being easily accessible and do not require invasive methods to obtain them, which is an important feature of the ideal biomarker. For this reason, over the last decades, several studies have been conducted to discover new biomarkers in biofluids that are derived from different pathological mechanisms of ALS [18,19,20,21,22]. The best considered biomarkers candidates are inflammatory molecules, metabolic markers and neurofilaments (NFs) [23,24]. 

To date, NFs are the most promising biomarkers for ALS. Particularly, both NF heavy chain (NFH) and NF light chain (NFL) levels measured in CSF and blood samples can be used to differentiate ALS patients from healthy subjects and/or other neurological diseases [25]. Furthermore, NFH and NFL levels in CSF were negatively correlated with disease duration, which address NFs as potential biomarkers for both diagnosis and prognosis of ALS [25]. 

Regarding inflammatory mediators, large panels of cytokines, including numerous interleukins, and immune cells, such as T regulatory cells (Treg), have been identified in CSF, plasma or serum, and have been correlated with faster or slower progression [26,27,28]. Cytokines and other inflammatory proteins will be discussed in depth in the following sections. 

The study of metabolic alterations in ALS patients is an increasing field of knowledge and more studies are needed to identify more reliable biomarkers related to metabolic changes in different tissues, such as CSF, blood and muscle [29,30]. For example, findings about glutamate levels in CSF as a metabolic biomarker of disease progression in patients remain controversial [19]. Nevertheless, in plasma samples, glutamate levels were increased and correlated with the disease duration and with patients that exhibited spinal onset [31,32]. In addition, glutamate levels can be used as a metabolic biomarker in serum in response to drug intervention [33]. On the other hand, mass spectrometry has allowed the identification of different metabolic profiles where several amino acids have shown different levels in blood and CSF of ALS patients compared to their controls; these metabolites include argine, lysine, serine and leucine, among others [19]. Other metabolites to consider are creatine and creatinine, which have been found dysregulated in CSF and blood [19,32], and used as molecular targets in a clinical trial [34]. Although altered metabolic profiles have been described in plasma samples of ALS patients, more reliable biomarkers that can contribute to disease progression and survival need to be identified [19].

microRNAs (miRNAs) are other potential biomarkers mainly due to their remarkably stability in body fluids. They have been found altered in CSF, plasma and serum from ALS patients and the SOD1G93A mouse model, such as MIR206, MIR143-3p, MIR338-3p [35,36,37,38]. Although some of these miRNAs are not specific for ALS, such as MIR206, it has been suggested that their combination could form part of a more integrative approach to help with ALS diagnosis and prognosis [38].

In urine, only one biomarker with prognostic value for ALS has been described, the extracellular domain of p75 neurotrophin receptor (p75^ECD^), whose levels showed an association with disease progression [39]. 

In spite of the fact that numerous potential biomarkers have been identified so far, none of them separately has sufficient sensitivity or specificity to become a universal biomarker for ALS. The lack of a robust biomarker is also reflected by some contradictory results and inconsistencies found in the literature, possibly due to patient heterogeneity and the complex scenario comprising this disease. 

## 3. The Role of The Immune System in ALS

The dysregulation of the immune system in ALS results in increased central and peripheral inflammatory responses [40]. Neuroinflammation is characterized by microglial activation, astrogliosis, infiltration of T lymphocytes and monocytes, and overproduction of inflammatory cytokines [41,42,43]. Both innate and adaptive immune responses are involved in ALS progression and can promote either neuroprotection or neurotoxicity depending on disease stage, evidencing a dual role of inflammation in ALS [44]. Initially, there is an early anti-inflammatory or neuroprotective phase, where neurotrophic factors and anti-inflammatory cytokines, such as interleukin (IL)-4 and IL-10, are secreted by surrounding astrocytes and M2 microglia. As the disease progresses, the neuroprotective response changes to a cytotoxic phase due to the activation of M1 microglia and the consequent release of toxic factors, including reactive oxygen species (ROS) and pro-inflammatory cytokines, such as IL-1β and tumor necrosis factor (TNF)-α, which causes progressive injury to the MN [45].

Apart from microglia and astrocytes, T lymphocytes of the adaptive immune response also play a relevant role in the neurodegeneration observed in ALS. Especially, CD4+ T helper (Th) lymphocytes, including Th2 lymphocytes and Treg, are found in the early neuroprotective compensatory response; on the contrary, CD8+ T cytotoxic lymphocytes, such as Th1 and Th17 lymphocytes, are observed at later stages of the disease [45,46]. Both types of T lymphocytes have shown a dual role: Th1 and Th17 expression was found elevated in blood from ALS patients [47], whereas an upregulation of Treg in blood was associated with slower progression of the disease [48]. Similarly, increasing levels of Treg in animal models at early stages in the disease prolonged survival [49].

Other cells involved in MN degeneration are monocytes and macrophages. They have been found activated in peripheral blood and in CNS, especially in the spinal cord, due to the existent disruption of the blood-spinal cord barrier, in both ALS patients and murine models [50,51]. In line with this, some components of the complement system, which participate in the recruitment of mononuclear cells and macrophages, have been found elevated in CSF, blood, spinal cord and skeletal muscle from ALS patients or murine models [42,52,53]. However, the role of the complement system in ALS pathogenesis remains controversial due to the diverging results based on the great inter-individual differences during disease progression, although aberrant activation of the complement system is suggested to be involved in the pathophysiology in ALS animal models and patients [52]. 

More recently, the relevant role of the mutation C9orf72 in myeloid cells opened the door to altered microglial function that can link the connection between autoimmunity and ALS/FTD. Some studies on heterozygous C9orf72 (C9orf72+/–) mice have suggested an altered myeloid cell function and systemic immunity. Accordingly, similar immunological consequences have been observed in ALS patients. Therefore, the loss of function of C9orf72 together with a combination of mutations of ALS/FTD genes could promote neurodegeneration [54]. Finally, the consequence of an altered microglia in both mutant SOD1 mice and patients is an amplified generation of pro-inflammatory cytokines that can trigger TNF-α-mediated apoptosis [55].

Despite the diverse humoral and cellular factors being found to be dysregulated, supporting the evident role of neuroinflammation in ALS pathology, to date it is still unclear how these inflammatory mediators can influence the progression of the disease and how they can be helpful in the diagnosis of ALS.

## 4. Dysregulation of Cytokines in Biofluids

A high number of studies have been conducted to find dysregulated cytokines in CSF and blood that can become potential biomarkers for ALS, mainly involving ALS patients, although few studies have been also performed in ALS animal models. 

### 4.1. Interleukins

Interleukins comprise of a large family of cytokines that can exert both pro-inflammatory and anti-inflammatory actions. They are mainly synthesized by T cells, macrophages and endothelial cells promoting the development and differentiation of T and B cells, and hematopoietic cells. Numerous interleukins have been found elevated in CSF and/or blood from ALS patients compared to the levels measured in controls and/or patients with other non-inflammatory neurological disorders (OND): IL-1Ra, IL-1β, IL-2, IL-4, IL-5, IL-6, IL-7, IL-8, IL-9, IL-10, IL-12p70, IL-13, IL-15, IL-17, IL-17A, IL-18 and IL-21 [26,47,56,57,58,59,60,61,62,63,64,65,66,67,68,69,70] (Table 1). Some of these interleukins follow the same pattern in the SOD1G93A model. Increasing levels of IL-2, IL-6, IL-10, IL-13 and IL-17B R in the transgenic (TG) mice are observed at an early stage of the disease [71]. In contrast, few studies reported decreasing levels of circulating IL-2, IL-5, IL-6 and IL-10 in ALS patients [58,63,72], whereas others found no significant differences in the levels of IL-2 and IL-6 in CSF or blood between ALS patients and their controls [73,74]. 

Regarding the potential role of interleukins as prognostic biomarkers, rising levels of IL-4, IL-6 and IL-13 in CSF or blood from ALS patients were associated with disease progression and ALSFRS-R score [26,56,64] (Table 1). Additionally, levels of IL-2 in plasma correlated with poorer survival in both SOD1G93A mice and ALS patients [26,71]. In the animal model SOD1G93A, other interleukins found in plasma, IL-6 and IL-17B R, have been related to faster or slower progression of the disease [71]. 

Despite the large number of interleukins that seems to participate in the interplay of neuroinflammation in ALS pathogenesis, there is no definitive mediator that is being used in the clinical setting to help in diagnosis or predicting progression.

### 4.2. Tumor Necrosis Factors

TNF-α is a major pro-inflammatory cytokine. It is primarily secreted by activated macrophages and is involved in the induction of cytokine production, phagocyte cell activation, activation or expression of adhesion molecules, and growth stimulation [75]. In regards to ALS, increased TNF-α levels have been found in CSF and blood from ALS patients [26,60,65,68,69] (Table 2). On the contrary, additional studies have shown lower levels TNF-α in blood from ALS patients [72]. Furthermore, in some cases, TNF-α was not found altered in serum of ALS patients compared to controls [73] or its levels in neither CSF nor blood did not correlate with the severity and progression of the disease [74]. Due to these controversial results, its function in ALS pathogenesis is still uncertain likely due to its pleiotropic actions, as it can act in both pro- and anti-inflammatory responses [76]. 

Although TNF-α is the most studied factor of this family, other TNFs have been found dysregulated in ALS. For instance, blood levels of TNF Receptor Superfamily Member (TNFRSF)1A (CD120) were higher in ALS patients than controls [68,72] (Table 2). In the same line, other TNFs, including TNFRSF8 L, TNFRSF19 and TNF Superfamily Member (TNFSF)11, were elevated, and TNFRSF18 was reduced in the murine model SOD1G93A compared to the levels of WT mice at early stages of the disease [71]. In addition, the prognostic value of some of these factors has been evaluated, resulting in the association of high levels of TNFRSF18 and TNFRSF19 with poorer survival [71]. 

### 4.3. Interferons (IFN)

The only circulating IFN found dysregulated in ALS patients to date is IFN-γ. This cytokine takes part in both innate and adaptive immunity by participating in the activation of macrophages and in the adaptive T cell response [28]. In ALS patients, IFN-γ has been found elevated in both CSF and blood compared to the levels measured in controls and OND, and have been associated with faster progression and shorter survival [47,60,67,77] (Table 3). In contrast, other studies suggested lower levels of this factor in CSF and blood from ALS patients [26,58], which questions its role as a potential biomarker for ALS. 

### 4.4. Colony Stimulating Factors (CSFs)

CSFs are responsible for stimulating proliferation and maturation of myeloid precursors. In particular, two of them have been linked to ALS: granulocyte (G)-CSF, involved in granulocyte production, and granulocyte-macrophage (GM)-CSF, participating in granulocyte, monocyte and eosinophil production. Both have been found increased in ALS patients in CSF and blood [58,60,64,67,69] (Table 4). In addition, levels of GM-CSF in blood negatively correlated with the duration of symptoms, which could help in the prognosis of the disease [78]. However, another study reported no significant differences in G-CSF levels in serum between ALS patients and controls or other neuropathies [73].

### 4.5. Chemokines

The main function of pro-inflammatory chemokines is to direct immune cells to the site of inflammation via chemotaxis. As interleukins, this family encloses a vast group of both chemokines and chemokine receptors. Regarding ALS, increased expression of several members of chemokines was found in CSF or blood from ALS patients than in controls or OND: C–C motif chemokine ligand (CCL)2, CCL3, CCL4, CCL11, C-X-C motif chemokine ligand (CXCL)8 and CXCL10; in addition, their levels also correlated with ALSFRS-R score or disease progression rate (DPR) [57,58,60,64,67,69,78,79,80] (Table 5). In the case of CCL5, higher levels were also found in ALS patients in CSF [61], but not in blood, where its levels, together with CXC5R, were lower than in controls [72]. 

Similarly in the SOD1G93A model, four chemokines were upregulated at early stages in TG mice, CCL3, CCL11, CCL19 and CCL21, suggesting a dysregulation of these mediators before the onset of symptoms [71] (Table 5). In addition, CCL3 and CCL11 negatively correlated with the longevity of TG mice. 

### 4.6. Other Cytokines and Proteins Related to Inflammation

Other proteins related to inflammation have been linked to ALS pathogenesis showing modified levels between ALS patients and their controls, and during disease progression (Table 6). Basic fibroblast growth factor (bFGF) and platelet-derived growth factor (PDGF)-BB were found elevated in CSF or blood in ALS patients [58,64,67]. On the contrary, other studies reported an association of high levels of bFGF with slower progression of the disease, which could be contradictory [67].

In the case of vascular endothelial growth factor (VEGF), higher levels were described in blood and CSF from ALS patients than in controls, whereas positive correlation with ALSFRS-R score and disease duration was reported, supporting the potential role of VEGF for ALS prognosis [58,60,67,68,69,81]. Low levels were also found in CSF from ALS patients during the first year of the disease [82]. In addition, an analysis combining VEGF with pNFH suggested a higher diagnostic yield [83]. 

Another cytokine altered in ALS is transforming growth factor beta (TGF-β), which is enrolled in the inhibition of T and B cell proliferation, hematopoiesis, and promotion of wound healing. In this case, higher levels were associated with faster progression of the disease in an ALS mouse model [84]. However, no differences were found in TGF-β levels in plasma from ALS patients [72]. 

Other proteins have also been shown to be altered in the SOD1G93A mouse model: activin receptor-like kinase (ALK)-1, galectin-1 and VEGF-D were elevated in plasma of TG mice [71]. In addition, rising levels of ALK-1 and galectin-1 in TG mice were associated with shorter survival [71]. In contrast, lower levels of galectin-3 in plasma were found in fast-progressing TG mice [84]. 

Interestingly, immunoglobulin G (IgG) antibodies have shown a relevant role for ALS prognosis. For instance, it has been reported that frequency and quantity of an IgG glycan is dependent on the ALS clinical stage in ALS animal models [85]. On the other hand, an increase in CSF IgG of the level of galactosylated structures was reported in ALS patients, showing a potential predictive value in the ROC analysis [86].

## 5. Cytokines as Biomarkers: Main Challenges

Over the last decades, many CSF and blood inflammatory cytokines have been found dysregulated in ALS, supporting the relevant contribution of neuroinflammation in the pathogenic mechanisms leading to motor neuron degeneration in ALS. However, the results shown are not always consistent in all the studies performed, which hampers the translation of a single cytokine as a biomarker to the clinical practice. As an example, it has been reported that the levels of certain cytokines in plasma from ALS patients were highly variable between the first and the second visit to the clinic, and even they did not show differences with healthy controls in some cases [63], exposing the great heterogeneity of the disease. In an attempt to deal with this issue, some authors have proposed that it would be more appropriate to identify panels of biomarkers, rather than focusing on a single target [87,88]. In this sense, panels of cytokines have been analyzed to help in a more accurate prediction of disease progression [89,90]. However, some authors suggest that these promising multivariate models should also include other clinical parameters, such as ALS type (sporadic or familial), disease stage, anatomical onset of motor neuron impairment and even age and gender [26,91]. 

Another problem that appears in the searching of circulating cytokines as biomarkers is the influence exerted by the action of environment and other factors surrounding the patient. For instance, the upregulation of some circulating cytokines, such as IL-6, IL-8, IL-10, G-CSF and TNF-α, has been linked to exercise [92], and others, including IL-6 and TNF-α, are elevated in a hypoxia status [93], which is a feature frequently found in ALS patients. In addition, the cytokine levels measured in blood in healthy individuals are not stable markers [94]. Cytokines play a relevant role in the immune system, and alterations in this system due to infections, injuries, tissue trauma or inflammation, which is inherent in ALS, can unbalance the immune system, even more under neurodegenerative conditions. This imbalance can promote an intra-individual variation that could explain the high variability of cytokine levels observed in ALS [40,94]. Furthermore, in the context of ALS and FTD, the cytokine profile in blood is also challenging to interpret due to the disease state, environmental factors, and genetic background of the individuals that can lead to controversial results [40].

Therefore, the consideration of circulating cytokines and other circulating targets as biomarkers is being increasingly questioned, mainly due to the opposing and irreproducible results that have been shown in different studies [95]. Additionally, the underlying causes, such as lack of sensitivity, unsuitable normalization or variations in sample handling, together with the difficulties in cytokine assays that are not performed in routine clinical methodology, establish this issue as a real challenge. In an attempt to address this problem, Otto et al. proposed a roadmap for biomarker discovery and provided standard operating procedures that could allow multicenter collaboration and validation of the neurochemical markers discovered in ALS to facilitate their translation to the clinic [96]. In this line, multicenter studies can also shed light on this controversial issue by confirming the results among different centers in the world, as demonstrated in several multicenter studies performed with ALS patients [97,98,99]. Accordingly, it could be interesting to contemplate multicenter studies regarding the most promising inflammatory mediators, which could be helpful to validate them.

## 6. Design of Therapeutic Approaches Targeting Inflammation

Given the number of cytokines found dysregulated in ALS, in the last decades many studies have translated the focus in assessing the potential role of those cytokines in therapies counteracting inflammation in ALS. In fact, there are currently several clinical trials involving therapies targeting neuroinflammation in ALS [42]. G-CSF is one of the targets that have provided beneficial results when administrated in both animal models and patients [100,101,102]. Similarly, other studies have shown that the administration of IL-33 or IFN-γ antibody delayed the disease onset and the motor decline, respectively, in an ALS mouse model [103,104]. However, there are also controversial results when translating the findings obtained in the animal model to ALS patients. For example, therapies using thalidomide and lenalidomide to inactivate TNF-α prolonged lifespan and enhanced the motor performance in the animal model [105,106]; in contrast, thalidomide treatment did not cause any improvement when administrated to ALS patients [107]. Regarding IL-6, transgenic mice deficient in IL-6 did not show any improvement, as well as a blocking IL-6 therapy in an ALS mouse model [108,109]. In view of this, it seems evident that therapies targeting a single factor could not provide meaningful benefits in ALS patients, as well as in the search for a reliable biomarker. Hooten et al. address questions on why these kinds of therapies are failing, highlighting that it could be too late when they are applied, or that therapies might not hit appropriate targets [45].

Recently, cell-based therapies are emerging as a promising strategy to modulate the immune system in ALS as they can influence different key immune targets. For instance, experimental treatments based on mesenchymal stem cells (MSCs) have shown to raise concentrations of neurotrophic factors, and elevate anti-inflammatory cytokines, which reduces neuroinflammation [43,110]. On the other hand, therapies targeting Treg, which were associated with disease progression [27,111], have also shown interesting results in ALS patients [112]. 

## 7. Conclusions 

ALS is a multifactorial disease where different pathological mechanisms direct or indirectly contribute to the degeneration of motor neurons. Neuroinflammation is one of these mechanisms investigated, since the involvement of many inflammatory mediators in this disease has been widely reported. However, the identification of specific cytokines to help in the diagnosis and also to predict the progression of the disease in ALS patients is challenging due to the great heterogeneity found in this disease. In addition, cytokines are variable and susceptible to the disease stage and to environmental factors surrounding ALS patients, who do not show an equal status of neuroinflammation. Consequently, the different pro and anti- inflammatory cytokines along the disease progression should be further studied to understand its time point activation and its relation to other molecular and clinical mediators in ALS to finally provide a better monitorization of disease progression. In this sense, cytokines could be helpful in improving the stratification of ALS patients according to their inflammatory status, enabling more accurate therapeutic approaches targeting these key immune factors.

## Figures and Tables

**Table 1:** Interleukins (IL) found dysregulated and proposed as biomarkers in biofluids from ALS animal models and patients.

Cytokine	Subject	Biofluid	Significance	Reference
**IL-1Ra**	Patients	Blood	Higher in ALS patients than in OND	[61]
**IL-1β**	Patients	CSF, blood	Higher in ALS patients than controls and/or OND	[26,60,68]
**IL-2**	Mouse model	Blood	Higher in TG mice than WT mice	[71]
			Negative correlation with longevity	[71]
	Patients	CSF, blood	Higher in ALS patients than controls and/or OND	[26,58,67,69,70]
		Blood	Lower in ALS than healthy controls	[63]
			Predictor of poor survival	[26]
**IL-4**	Patients	CSF, blood	Higher in ALS patients than controls and/or OND	[26,64]
		CSF	Higher levels associated with slower disease progression	[64]
**IL-5**	Patients	Blood	Higher in ALS patients than healthy controls	[26]
			Lower in ALS patients than healthy controls	[63]
**IL-6**	Mouse model	Blood	Higher in TG mice than WT mice	[71]
			Negative correlation with longevity	[71]
	Patients	CSF, blood	Higher in ALS patients than controls and/or OND	[26,47,58,63,65,66,67,68,70]
		Blood	Lower in ALS patients than controls	[72]
		Blood	Rising levels associated with disease progression	[26]
**IL-7**	Patients	CSF	Higher in ALS patients than in OND	[60,64]
**IL-8**	Patients	CSF, blood	Higher in ALS patients than controls and/or OND	[26,57,63,65,66,68,70]
**IL-9**	Patients	CSF	Higher in ALS patients than in OND	[60]
**IL-10**	Mouse model	Blood	Higher in TG mice than WT mice	[71]
	Patients	Blood	Higher in ALS patients than controls	[26,67]
		CSF, blood	Lower in ALS patients than controls and/or OND	[58,72]
		CSF	Higher levels associated with milder symptoms	[64]
**IL-12p70**	Patients	CSF, blood	Higher in ALS patients than controls and/or OND	[26,60]
**IL-13**	Mouse model	Blood	Higher in TG mice than WT mice	[71]
	Patients	Blood	Higher in ALS patients than controls	[26,56]
			Negative correlation with ALSFRS-R score	[56]
			Positive correlation with the DPR	[56]
**IL-15**	Patients	CSF, blood	Higher in ALS patients than controls and/or OND	[58,67,69]
**IL-17**	Patients	CSF, blood	Higher in ALS patients than controls and/or OND	[47,58,60,64,67,69]
**IL-17A**	Patients	Blood	Higher in ALS than controls	[59]
**IL-17B R**	Mouse model	Blood	Higher in TG mice than WT mice	[71]
			Higher levels associated with shorter survival	[71]
**IL-18**	Patients	Blood	Higher in ALS patients than controls	[62]
**IL-21**	Patients	Blood	Higher in ALS patients than controls	[47]

**Table 2:** Tumor necrosis factors (TNF) found dysregulated and proposed as biomarkers in biofluids from ALS animal models and patients.

Cytokine	Subject	Biofluid	Significance	Reference
**TNF-α**	Patients	CSF, blood	Higher in ALS patients than controls and/or OND	[26,60,65,68,69]
		Blood	Lower in ALS patients than controls	[72]
**TNFRSF1 (CD120)**	Patients	Blood	Higher in ALS patients than controls	[68,72]
**TNFRSF8 L (CD30 L)**	Mouse model	Blood	Higher in TG mice than WT mice	[71]
**TNFRSF18 (GITR)**	Mouse model	Blood	Lower in TG mice than WT mice	[71]
			Higher levels associated with shorter survival	[71]
**TNFRSF19 (TROY)**	Mouse model	Blood	Higher in TG mice than WT mice	[71]
			Negative correlation with longevity	[71]
**TNFSF11 (RANKL)**	Mouse model	Blood	Higher in TG mice than WT mice	[71]

**Table 3:** Interferons (IFN) found dysregulated and proposed as biomarkers in biofluids from ALS patients.

Cytokine	Subject	Biofluid	Significance	Reference
**IFN-γ**	Patients	CSF, blood	Higher in ALS than controls and/or OND patients	[47,60,67,77]
			Lower in ALS than controls or OND patients	[26,58]
			Positive correlation with DPR	[67,77]
		CSF	Higher levels associated with shorter overall survival.	[67]

**Table 4:** Colony stimulating factors (CSFs) found dysregulated and proposed as biomarkers in biofluids from ALS patients.

Cytokine	Subject	Biofluid	Significance	Reference
**G-CSF**	Patients	CSF, blood	Higher in ALS patients than controls and/or OND	[58,60,64,67,69]
**GM-CSF**	Patients	CSF, blood	Higher in ALS patients than controls and/or OND	[58,67]
		Blood	Negative correlation with duration of symptoms	[78]

**Table 5:** Chemokines found dysregulated and proposed as biomarkers in biofluids from ALS animal models and patients.

Cytokine	Subject	Biofluid	Significance	Reference
**CCL2 (MCP-1)**	Patients	CSF, blood	Higher in ALS patients than controls and/or OND	[57,58,60,67,69,78,80]
			Negative correlation with ALSFRS-R score	[60,67]
			Positive correlation with DPR	[67]
		Blood	Negative correlation with duration of symptoms	[78]
**CCL3 (MIP-1α)**	Mouse model	Blood	Higher in TG mice than WT mice	[71]
			Negative correlation with longevity	[71]
	Patients	CSF, blood	Higher in ALS patients than controls and/or OND	[58,67,69,79]
			Positive correlation with disease duration and negative correlation with DPR	[67,79]
**CCL4 (MIP-1β)**	Patients	CSF	Higher in ALS patients than controls and OND	[58,60,67,80]
			Positive correlation with ALSFRS-R and disease duration, and negative correlations with DPR	[60]
**CCL5 (RANTES)**	Patients	CSF	Higher in ALS patients than in OND	[60]
		Blood	Lower in ALS patients than controls	[72]
**CCL11 (Eotaxin-1)**	Mouse model	Blood	Higher in TG mice than WT mice	[71]
			Higher levels associated with shorter survival and negative correlation with longevity	[71]
	Patients	CSF, blood	Higher in ALS patients than in OND	[57,60,64]
		CSF	Higher levels with slower DPR	[64]
**CCL19 (MIP-3β)**	Mouse model	Blood	Higher in TG mice than WT mice	[71]
**CCL21 (6Ckine)**	Mouse model	Blood	Higher in TG mice than WT mice	[71]
**CXC5R**	Patients	Blood	Lower in ALS patients than controls	[72]
**CXCL8**	Patients	CSF	Higher in ALS patients than in OND	[60]
			Negative correlation with ALSFRS-R score	[60]
**CXCL10**	Patients	CSF	Higher in ALS patients than in OND	[60]
			Negative correlation with DPR	[60]

**Table 6:** Other cytokines and proteins related to inflammation found dysregulated and proposed as biomarkers in biofluids from ALS animal models and patients.

Protein	Subject	Biofluid	Significance	Reference
**ALK-1**	Mouse model	Blood	Higher in TG mice than WT mice	[71]
			Higher levels associated with shorter survival	[71]
**bFGF**	Patients	CSF, blood	Higher in ALS patients than controls and/or OND	[58,64,67]
			Positive correlation with disease duration	[67]
		Blood	Negative correlation with DPR	[67]
**Galectin-1**	Mouse model	Blood	Higher in TG mice than WT mice	[71]
			Higher levels associated with shorter survival and negative correlation with longevity	[71]
**Galectin-3**	Mouse model	Blood	Lower in fast-progressing TG mice at pre-symptomatic and symptomatic than in WT mice	[84]
**IgG**	Patients	CSF	Increased level of galactosylated structures	[86]
**PDGF-BB**	Patients	CSF	Higher in ALS patients than in OND	[64]
**TGF-β**	Mouse model	Blood	Higher in slow-progressing TG mice and lower in fast-progressing TG mice at pre-symptomatic and symptomatic than in WT mice	[84]
**VEGF**	Patients	CSF	Low levels at early stages of ALS	[82]
		CSF, blood	Higher in ALS patients than controls and/or OND	[58,60,67,68,69]
			Positive correlation with ALSFRS-R score and disease duration	[60,67,81]
			Negative correlation with DPR	[81]
**VEGF-D**	Mouse model	Blood	Higher in TG mice than WT mice	[71]

## Abbreviations

ALK-1	Activin receptor-like kinase 1
ALS	Amyotrophic lateral sclerosis
ALSFRS-R	Amyotrophic lateral sclerosis functional rating scale-revised
bFGF	Basic fibroblast growth factor
C9ORF72	Chromosome 9 open reading frame 72
CCL	C-C motif chemokine ligand
CSF	Cerebrospinal fluid
CSFs	Colony stimulating factors
CXCL	C-X-C motif chemokine ligand
CXC5R	C-X-C motif chemokine receptor 5
DPR	Disease progression rate
DTI	Diffusion tensor imaging
FUS	Fused in sarcoma
G-CSF	Granulocyte colony stimulating factor
GITR	Glucocorticoid-induced TNFR-related protein
GM-CSF	Granulocyte-macrophage colony stimulating factor
IL	Interleukin
IFN	Interferons
IgG	Immunoglobulin G
MCP-1	Monocyte chemotactic protein-1
miRNAs	microRNAs
MIP	Macrophage inflammatory protein
MN(s)	Motor neuron(s)
MRI	Magnetic resonance imaging
MRS	Magnetic resonance spectroscopy
NFs	Neurofilaments
NFH	Neurofilament heavy chain
NFL	Neurofilament light chain
OND	Other non-inflammatory neurological disorders
p75^ECD^	Extracellular domain of p75 neurotrophin receptor
PDGF-BB	Platelet-derived growth factor BB
PET	Positron emission tomography
RANKL	Receptor activator of nuclear factor kappa-Β ligand
ROS	Reactive oxygen species
SOD1	Superoxide dismutase 1
TDP-43	TAR DNA binding protein 43
TGF-β	Transforming growth factor beta
TG	Transgenic
Th	T helper
TMS	Transcranial magnetic stimulation
TNF	Tumor necrosis factor
TNFRSF	TNF receptor superfamily member
TNFSF	TNF superfamily member
Treg	Regulatory T cell
TROY	Tumor necrosis factor receptor superfamily member 19
VEGF	Vascular endothelial growth factor
WT	Wildtype
